# Identification of the Transgene Integration Site and Host Genome Changes in MRP8-Cre/ires-EGFP Transgenic Mice by Targeted Locus Amplification

**DOI:** 10.3389/fimmu.2022.875991

**Published:** 2022-04-06

**Authors:** Guan Wang, Cunling Zhang, Hiroto Kambara, Cheryl Dambrot, Xuemei Xie, Li Zhao, Rong Xu, Andrea Oneglia, Fei Liu, Hongbo R. Luo

**Affiliations:** ^1^ Department of Pathology, Dana-Farber/Harvard Cancer Center, Harvard Medical School, Boston, MA, United States; ^2^ Department of Laboratory Medicine, Enders Research Building, Boston Children’s Hospital, Boston, MA, United States; ^3^ The State Key Laboratory of Experimental Hematology, Institute of Hematology and Blood Diseases Hospital, Chinese Academy of Medical Sciences and Peking Union Medical College, Tianjin, China; ^4^ Cergentis BV, Utrecht, Netherlands

**Keywords:** MRP8-Cre transgenic mouse, TLA sequencing, Cre-*loxP* system, neutrophil, homozygous lethality

## Abstract

The MRP8-Cre-ires/EGFP transgenic mouse (Mrp8cre^Tg^, on C57BL/6J genetic background) is popular in immunological and hematological research for specifically expressing Cre recombinase and an EGFP reporter in neutrophils. It is often crossed with other transgenic lines carrying *loxP*-flanked genes to achieve restricted gene knockout in neutrophils. However, due to the way in which the line was created, basic knowledge about the MRP8-Cre-ires/EGFP transgene in the host genome, such as its integration site(s) and flanking sequences, remains largely unknown, hampering robust experimental design and data interpretation. Here we used a recently developed technique, targeted locus amplification (TLA) sequencing, to fill these knowledge gaps. We found that the MRP8-Cre-ires/EGFP transgene was integrated into chromosome 5 (5qG2) of the host mouse genome. This integration led to a 44 kb deletion of the host genomic sequence, resulting in complete deletion of *Serpine1* and partial deletion of *Ap1s1*. Having determined the flanking sequences of the transgene, we designed a new genotyping protocol that can distinguish homozygous, heterozygous, and wildtype Mrp8cre^Tg^ mice. To our surprise, crossing heterozygous mice produced no homozygous Mrp8cre^Tg^ mice, most likely due to prenatal lethality resulting from disrupted *Ap1s1* gene expression.

## Introduction

Cells of myeloid lineage play crucial roles in maintaining tissue homeostasis and integrity, hematopoiesis, and innate/adaptive immune responses (1–4). Therefore, myeloid cells such as neutrophils, monocytes, and macrophages have become popular targets for cell type-specific gene manipulation in new transgenic animal models (5–9). The Cre-*loxP* system is a powerful genome-editing tool capable of introducing deletions, inversions, and translocations of DNA fragments by flanking target genes with *loxP* sequences (10–13). The Cre-*loxP* system can also be used for conditional gene inactivation in certain tissues or cell types through specific gene promoter-driven Cre recombinase expression, thereby enabling the investigation of gene function in specific and carefully defined contexts (14–17).

Myeloid-related protein 8 (MRP8), also known as S100A8 in humans, is a well characterized calcium-binding protein specifically expressed in neutrophils and monocytes (18, 19). In transgenic mice expressing human *MRP8* (*hMRP8*), the majority of *hMRP8* promoter-driven B cell lymphoma-2 (BCL-2) expression was in neutrophils (7). Based on this observation, another transgenic mouse line, MRP8-Cre-ires/EGFP (or Mrp8cre^Tg^), was generated to specifically express Cre recombinase and the enhanced green fluorescent protein (EGFP) reporter protein in neutrophils. The Mrp8cre^Tg^ mouse carries a transgene containing a Cre/ires-EGFP construct placed downstream of the *hMRP8* promoter introduced randomly into the mouse genome *via* microinjection of fertilized oocytes (8). The Mrp8cre^Tg^ transgenic mouse line has since been used extensively in studies of neutrophil function under pathophysiological conditions (20–24).

Nevertheless, despite its popularity, the random integration of the MRP8-Cre-ires/EGFP transgene through oocyte microinjection means that little is known about its genetic context. Therefore, in this study, we utilized the targeted locus amplification (TLA) technique to uncover the precise location, flanking sequences, and potential impact on the host genome of MRP8-Cre-ires/EGFP transgene. TLA is a recently developed sequencing strategy that can selectively amplify and sequence the entire genes based on crosslinked proximal DNA sequences in genome without knowing the detailed information of the target genes and the flanking regions (25). It is hence particularly suitable for revealing the transgene information of Cre transgenic mice lines which were generated *via* traditional techniques such as oocyte microinjection (26). Gained knowledge of the details of the transgene insertion into the host genome would be helpful for genotyping, experimental design, and accurate interpretation of results. This information subsequently allowed us to design a new genotyping protocol that can distinguish homozygous, heterozygous, and wildtype Mrp8cre^Tg^ mice. Surprisingly, no homozygous offspring were observed when mating heterozygous Mrp8cre^Tg^ mice, presumably due to disrupted expression of *Ap1s1*.

## Methods

### The MRP8-Cre-ires/EGFP Transgenic Mouse

The MRP8-Cre-ires/EGFP (Mrp8cre^Tg^) transgenic mouse line was originally created in Dr. Irving L. Weissman’s laboratory before being deposited in The Jackson Laboratory (stock #021614; Bar Harbor, ME). The mouse was designed to specifically express Cre recombinase and the EGFP reporter protein in myeloid cells (mainly neutrophils and monocytes; see original paper for a detailed description) (8). Briefly, the hMRP8-Cre-ires/EGFP transgene was microinjected into the pronucleus of (C57BL/6 x C3H) F1 fertilized oocytes. The resulting transgenic mice were then backcrossed with C57BL/6 mice for two to seven generations before being used in experiments. This line was further bred with C57BL/6J mice for at least nine generations in The Jackson Laboratory (Strain No.: 021614). More detailed information on the breeding, maintenance, development, phenotypes, applications, and technical support can be found on the Jackson Laboratory website (https://www.jax.org/strain/021614). In a more recent study that assessed the knockout efficiency and specificity of the Cre-*loxP* system in myeloid cells, this transgenic line was found to produce the highest and most specific knockout effects in neutrophils (>80%) (21).

### Splenocytes Preparation

Two Mrp8cre^Tg^ mice (a male and a female, 8-week old) were euthanized with CO^2^ and the spleens were dissected and stored on ice. Each dissected spleen was then made into a single cell suspension in 0.5 ml phosphate buffered saline (PBS) by gently pressing it through a 40 *μ*m cell strainer. The splenocytes were then collected by centrifugation at 4°C, 500g for 5 min. The supernatant was discarded and the pellet was resuspended and incubated in 0.5 mL ACK lysis buffer (Gibco, A1049201) at 4°C for 3 min to lyse the splenic erythrocytes. To terminate the lysis reaction, 0.5 ml PBS was added and the splenocytes were collected by centrifugation at 4°C, 500g for 5 min. After centrifuging, the supernatant was discarded and the pellet was resuspended in 0.5 ml PBS again. After another 2 min centrifugation, the supernatant was discarded and cell pellet was resuspended in 1 mL freezing medium (PBS with 10% Dimethyl Sulfoxide and 10% fetal bovine serum). The samples were stored at -80°C until next step TLA processing.

### Targeted Locus Amplification (TLA) and Genomic DNA Sequencing

Here we use the TLA method to assess transgene fusion, integration site, and copy number in the mouse genome (25, 26) as well as single nucleotide variants (SNVs) and structural variants surrounding the transgene integration site(s). Viable frozen spleen cells from MRP8-Cre-ires/EGFP mice were prepared for TLA assessment following the manufacturer’s protocol (25) (Cergentis, Utrecht, Netherlands). Detailed description of the TLA sequencing sample preparation can be found in the original paper (25). Briefly, isolated splenocytes were crosslinked by formaldehyde and then the DNA was digested by enzyme NlaIII. Then the sample is ligated, crosslinks were reversed, and DNA was purified and trimmed with NspI and ligated at a DNA concentration of 5 ng/*μ*l in order to obtain circular chimeric DNA molecules for PCR amplification. NspI was chosen for its RCATGY recognition sequence that encompasses the CATG recognition sequence of NlaIII. As a consequence, only a subset of NlaIII (CATG) sites were digested, generating DNA fragments of approximately 2 kb and allowing the amplification of entire restriction fragments. After ligation, the DNA was purified, and eight 25-*μ*l PCR reactions, each containing 100 *n*g template, were pooled for sequencing. Two sets of primers (see **Supplementary Figure 1B** for details) targeting the transgenic Cre or EGFP sequences were used in individual TLA amplifications. PCR products were purified, and the library was prepared using the Illumina Nextera flex protocol and sequenced on an Illumina sequencer (Illumina, San Diego, CA).

### Bioinformatics/Sequence Alignment

Reads of DNA fragments from next-generation sequencing (NGS) were mapped to a reference mouse genome (mm10). Because TLA protocol leads to reshuffling of the genomic DNA sequences, reads mapping was performed using the customized TLA data analysis pipeline, which is based on the BurrowsWheeler Aligner’s Smith-Waterman Alignment (BWA-SW) algorithm, version 0.7.15-r1140 (27). This is a two-step process that ensures maximum ‘mappability’ of the TLA data. The reads are firstly mapped to the genome similarly to regular sequencing data. Secondly, unaligned sequences are digested *in silico* on the basis of the NlaIII site and remapped to the mouse genome. The resulting BAM files are a combination of the first and second mapping iterations. Although paired-end sequencing was performed, the paired-end information was not used in the mapping process owing to reshuffling of the sequences. Paired ends are therefore treated separately in the general analysis.

### Genotyping MRP8-Cre-ires/EGFP Transgenic Mice

Based on the TLA sequencing results, a new primer set was designed and tested to distinguish homozygous, heterozygous, and wildtype Mrp8cre^Tg^ mice. Genomic DNA was extracted from the tails or ears by incubating with 75 *μ*L NaOH DNA extraction buffer (25 mM NaOH; 0.2 mM EDTA) at 95°C for 45-60 minutes before being neutralized with an equal volume of 40 mM Tris HCl (pH 5.5). After centrifuging, 1 *μ*L of supernatant was taken for each PCR reaction with Q5 High-Fidelity 2X Master Mix (New England Biolabs, Ipswich, MA; M0492). The primer sequences were: forward: AGACAGGGTAGTAGCTCTGTGTAGC; reverse 1: GTGGAGGGACCTCAAAGTTGTCTATAAG; reverse 2: GCTCACTGTAGCCTCGAACAC. For each PCR reaction, 1 *μ*M of each primer was used for genotyping. The NEB Q5 High-Fidelity PCR protocol was used for the reaction (https://www.neb.com/protocols/2012/08/29/pcr-using-nebnext-high-fidelity-2x-pcr-master-mix-m0541) with the annealing temperature set at 68°C. Two PCR fragments of 230 bp and 574 bp, respectively, were observed for heterozygous mice, while only the 574bp fragment was observed for wildtype mice. A single band of the 230 bp fragment is expected for homozygous Mrp8cre^Tg^ mice, which we did not observe in this study.

### Single Cell Collection, Sequencing, and Data Analysis

Detailed experimental procedures for the single-cell sequencing datasets used in this study have been described by Xie et al. and Xu-Vanpala et al., respectively (28, 29). Briefly, for the Xie et al. dataset, c-Kit^+^ and Gr1^+^ cells from the bone marrow, peripheral blood, and spleen were sorted into PBS containing 0.05% BSA followed by the 10X Genomics Chromium single-cell sequencing protocol (28). For the Xu-Vanpala et al. dataset, CD45^+^ cells from mouse lungs were enriched by positive selection using a combination of biotinylated anti-CD45 antibodies and streptavidin MicroBeads (Miltenyi Biotec, Bergisch Gladbach, Germany), followed by 10X Genomics Chromium single-cell sequencing (29). The same standards and parameters for single-cell data processing (quality control, cell number cutoff, and normalization) were applied to both datasets, which can be found in the original publications. Single-cell clustering and gene expression plotting were further accomplished using *Seurat* in R (version 4.0.1). Different cell types were identified using specific cell markers.

## Results

### Structure of the hMRP8-Cre/ires-EGFP Transgene

The complete human *MRP8* (*S100A8*) gene was cloned and the specificity of MRP8 expression in myeloid cells established in 1988 (30). A Cre/ires-EGFP cloning cassette was then inserted into the *BglII* cutting site located between exons 2 and 3 (**Supplementary Figure 1A**) of the human *MRP8* gene to generate the hMRP8-Cre/ires-EGFP transgene. The 4.5 kb fragment between *HindIII* and *EcoRI* of *hMRP8* was used for transgene construction and transgenic mice generation (7, 8).

### Detecting the Insertion Site and Flanking Sequences of the hMRP8-Cre/ires-EGFP Transgene in the Mouse Genome by TLA Sequencing

**Figure 1:**
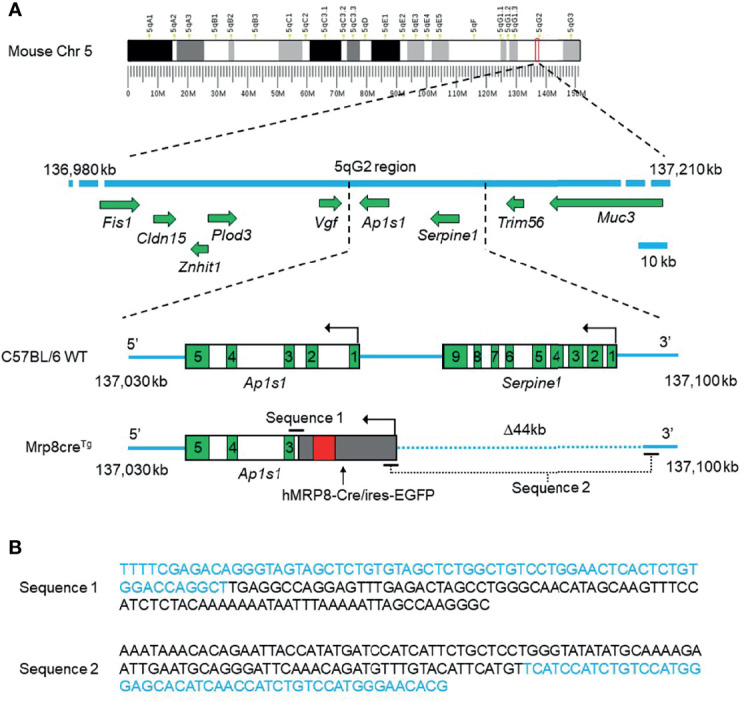
The integration site of the hMRP8-Cre/ires-EGFP transgene on mouse chromosome 5 (Chr 5) and the DNA sequences of the connecting regions between the mouse genome and human *MRP8*. The hMRP8-Cre/ires-EGFP transgene was integrated into the mouse genome within the intronic region of *Ap1s1* between exons 2 and 3 on chromosome 5, resulting in a 44 kb (Δ44 kb) deletion of the mouse genome containing the full sequence of *Serpine1* and a partial sequence of *Ap1s1*. **(A)** The schematic shows wild-type C57BL/6 mouse Chr 5 (the blue line) with intact *Ap1s1* and *Serpine1* and the altered Chr 5 in Mrp8cre^Tg^ mice with the inserted hMRP8-Cre/ires-EGFP transgene. The dotted blue line indicates a 44 kb deletion of the mouse genome on Chr 5 (cartoons not drawn to scale). Green boxes indicate exons. **(B)** DNA sequences of the connecting regions between the mouse genome and the integrated hMRP8-Cre/ires-EGFP transgene. Letters in blue indicate the mouse genomic sequence, while letters in black indicate the hMRP8-Cre/ires-EGFP transgenic sequence.

Due to its random insertion, the exact location and flanking sequences of the hMRP8-Cre/ires-EGFP transgene in the host genome remains unknown, so determining homozygosity and heterozygosity of Mrp8cre^Tg^ mice has previously been unfeasible. Here, Cergentis B.V. performed TLA sequencing to determine the precise location of the hMRP8-Cre/ires-EGFP transgene in the mouse genome as well as the connecting sequences between genes (25, 26). Two primer sets targeting the Cre or EGFP regions of the transgene, respectively, were designed for TLA sequencing (**Supplementary Figure 1B**). Genomic DNA extracted from viable frozen spleen cells of two Mrp8cre^Tg^ mice (a male and a female) were processed for sequencing following the Cergentis TLA protocol (25, 26). Reads of genomic DNA fragments were subsequently mapped to a reference mouse genome (mm10) and aligned subsequently. Sequence alignment showed that the hMRP8-Cre/ires-EGFP transgene was inserted in mouse chromosome 5 between 137,042,580 and 137,086,690 bp (5qG2 region, mm10 *Mus musculus* reference genome), leading to a 44 kb deletion of the host genome (**Figure 1A** and **Supplementary Figure 2**). This insertion site is located within the intronic region of *Ap1s1* (adaptor related protein complex 1 subunit sigma 1) between exon 2 and exon 3 (**Figure 1** and **Supplementary Figure 2B**). Similar results were obtained in both male and female mice. We were also able to determine the DNA sequences across the connecting regions of genes, including mouse *Ap1s1*-hMRP8, hMRP8-Cre, Cre-ires, ires-EGFP, and EGFP-hMRP8 (for the entire sequence reconstruction, see **Supplementary Figure 3**). In particular, the specific sequences spanning the mouse genome to the hMRP8 5’ promoter region and the hMRP8 3’ regulatory region to the mouse genome were detected (**Figure 1B**), enabling us to design a new set of primers for Mrp8cre^Tg^ genotyping.

### *Ap1s1* and *Serpine1* Gene Expression in Mouse Myeloid Cells

Given TLA sequencing suggested complete deletion of *Serpine1* and partial deletion of *Ap1s1* from the Mrp8cre^Tg^ mouse genome, it was important to quantify endogenous expression of these genes in myeloid cells. We utilized two available single-cell sequencing datasets, one from our previous study of bone marrow, peripheral blood, and spleen myeloid cells (GSE137540) (28) and another of mainly lung immune cells (GSE146233) (29). In both datasets, mRNA expression of *Ap1s1* and *Serpine1* was very low in mature neutrophils (**Supplementary Figures 4A, B**) but relatively high in alveolar macrophages (**Supplementary Figure 4B**). While *Serpine1* mRNA was not expressed in any other immune cells except for macrophages, *Ap1s1* was expressed at low to medium levels in different immune cell types including myeloid progenitors, dendritic cells (DC), monocytes, B cells, and T cells (**Supplementary Figures 5A, B**).

### Identifying Heterozygous Mrp8cre^Tg^ Mice With the New Genotyping Protocol

**Figure 2:**
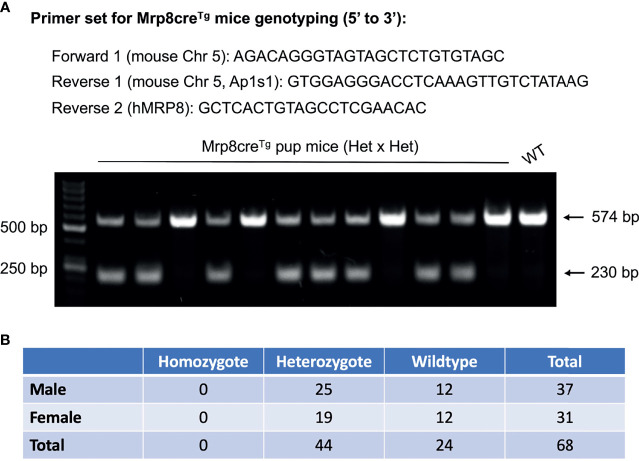
A new genotyping protocol distinguishes the different genotypes of Mrp8cre^Tg^ mice. **(A)** The primer set designed for Mrp8cre^Tg^ mouse genotyping based on TLA sequencing results (upper panel). A representative DNA gel genotyping two litters of Mrp8cre^Tg^ mice bred by mating male heterozygotes with female heterozygotes. Two DNA bands of 574 bp and 230 bp are observed for heterozygous pups, while only one 574 bp band is observed for wildtype pups (lower panel). **(B)** Amongst 68 Mrp8cre^Tg^ pups bred from heterozygous parents, 44 heterozygous versus 24 wildtypes were detected and no homozygous pups were found.

Due to the lack of information on transgene integration, previous Mrp8cre^Tg^ genotyping protocols have been generic and only detect the presence or absence of Cre, which does not distinguish homozygous from heterozygous mice (Jackson Lab protocol: 22392) (21). With the flanking sequences available, we designed a new set of primers that successfully detected heterozygous Mrp8cre^Tg^ mice and wildtypes (**Figure 2A**, **Supplementary Figure 5**). Surprisingly, however, there were no homozygous mice in 68 offspring reproduced by mating heterozygous Mrp8cre^Tg^ mice (**Figure 2B**). Since the ratio of Mrp8cre^Tg^ heterozygotes to wildtypes was close to 2:1, prenatal/preweaning lethality of Mrp8cre^Tg^ homozygotes is highly likely.

## Discussion

The Mrp8cre^Tg^ mouse is by far the most neutrophil-specific Cre line (>80%), and it has been extensively used in studies investigating neutrophil function (21). Abram et al. showed that crossing Mrp8cre^Tg^ strain with ROSA26-flox-stop-flox-EYFP reporter mice (ROSA-EYFP) resulted in over 80% Cre-mediated deletion in granulocyte populations from spleen, peripheral blood and bone marrow, which is the highest fidelity amongst all similar transgenic strains (21).When crossing Mrp8cre^Tg^ mice with other “floxed” transgenic lines, the simultaneous expression of both the Cre recombinase and EGFP reporter protein in neutrophils provides the advantage of being able to visualize cells with Cre-Lox recombination. To better understand the line and facilitate future studies, we performed TLA sequencing to determine the precise location and flanking sequences of the transgene in the host genome. Having determined the sequence, we were able to design a new genotyping protocol to detect homozygous and heterozygous Mrp8cre^Tg^ mice. Surprisingly, however, there were no homozygotes in 68 offspring produced by mating heterozygous Mrp8cre^Tg^ mice.

The hMRP8-Cre/ires-EGFP transgene was randomly integrated into chromosome 5 and resulted in a 44 kb deletion of the host mouse genome. As a result, the whole *Serpine1* gene and part of *Ap1s1* were deleted from the mouse genome, likely disrupting the expression of both genes. Both *Ap1s1* and *Serpine1* encode proteins exerting vital physiological functions. The *Ap1s1* gene encodes the small subunit of the adaptor related protein complex 1 subunit sigma 1 (AP1S1) (31, 32), which is part of the adaptor protein (AP) complexes regulating clathrin-coated vesicle assembly, receptor endocytosis, and Golgi processing (33). In humans, disrupted of *AP1S1* has been shown in association with MEDNIK (mental retardation, enteropathy, deafness, neuropathy, ichthyosis, keratodermia) syndrome (34–36). In another recent study, it was shown that the loss of *AP1S1* function caused by missense mutations leads to intestinal epithelial barrier defect, and a non-syndromic form of congenital diarrhea (37). In mice, according to data from International Mouse Phenotyping Consortium (IMPC), embryonic lethality is observed in homozygous *Ap1s1* knockout strain (*Ap1s1^tm1.1(KOMP)Vlcg^*, MGI ID: 1098244). Thus, it is plausible that the lack of homozygous Mrp8cre^Tg^ mice is due to the deletion of *Ap1s1* gene from the host genome. Deletion of gene *Serpine1* may also complicate the interpretation of the results of studies using Mrp8cre^Tg^ mice, given its roles in innate immunity and inflammatory response during infection (e.g. sepsis) (38–40). The protein encoded by *Serpine1*, PAI-1 (plasminogen activator inhibitor-1, aka. serpin E1), is a serine protease inhibitor that can suppress fibrinolysis, a physiological process of naturally breaking-down blood clots (41). PAI-1 is broadly expressed in platelets, neutrophils, macrophages, and other tissues, then secreted into the blood circulation (41, 42). Increased serum level of PAI-1 has been reported in a number of inflammatory diseases such as myocardial infarction, sepsis, and lung injury (43–45). PAI-1 directly affects innate immunity by mediating neutrophil homeostasis and activation under physiological or pathological conditions (46–50). In mice, deletion of *Serpine1* gene appears to induce a mild hyperfibrinolytic state and a greater resistance to venous thrombosis (51).

Due to the functional significance of these two genes, the potential impact of their deletion in experiments with Mrp8cre^Tg^ mice should be acknowledged and carefully controlled. Mrp8cre^Tg^ mice bearing the Cre recombinase gene are all heterozygotes, so a Cre mouse control group should be included in addition to a *LoxP* mouse control. Future studies using Mrp8cre^Tg^ mice should also consider the potential “low dose effect” produced by single copies of *Ap1s1* and *Serpine1*, in both *in vivo* and *in vitro* neutrophil functional assays. The heterozygous KO (Serpine1+/-Ap1s1+/-) may display different phenotypes in different systems under different settings.

## Data Availability Statement

The raw data supporting the conclusions of this article will be made available by the authors, without undue reservation.

## Ethics Statement

The animal study was reviewed and approved by Institutional Animal Care & Use Committee, Boston Children’s Hospital.

## Author Contributions

GW, CZ, FL, and HK performed the experiments. CD performed the TLA sequencing data analysis and prepared the original reports. XX performed the single-cell sequencing experiment. GW, FL, and CZ performed data analysis. LZ and RX prepared the animals needed in this study. GW drafted the manuscript with AO’s help. HL oversaw the study, designed experiments, and edited the manuscript for submission. All authors contributed to the article and approved the submitted version.

## Conflict of Interest

Authors CD and AO were employed by company Cergentis BV.

The remaining authors declare that the research was conducted in the absence of any commercial or financial relationships that could be construed as a potential conflict of interest.

## Publisher’s Note

All claims expressed in this article are solely those of the authors and do not necessarily represent those of their affiliated organizations, or those of the publisher, the editors and the reviewers. Any product that may be evaluated in this article, or claim that may be made by its manufacturer, is not guaranteed or endorsed by the publisher.
