# Supplementary material for: Identification of the Transgene Integration Site and Host Genome Changes in MRP8-Cre/ires-EGFP Transgenic Mice by Targeted Locus Amplification
Source: Front Immunol. 2022 Apr 6;13:875991. doi: 10.3389/fimmu.2022.875991 (PMC9020256; doi:10.3389/fimmu.2022.875991)
Supplement: Supplementary file 1 [file DataSheet_1.pdf]

Supplementary Material

Identification of the transgene integration site and host genome changes in MRP8-Cre/ires-EGFP transgenic mice by targeted locus amplification

Guan Wang[1]†, Cunling Zhang[2]†, Hiroto Kambara[1], Cheryl Dambrot[3], Xuemei Xie[1], Li Zhao[1], Rong Xu[1], Andrea Oneglia[3], Fei Liu[2], Hongbo R. Luo[1*]

[1]Department of Pathology, Dana-Farber/Harvard Cancer Center, Harvard Medical School; Department of Laboratory Medicine, Boston Children's Hospital, Enders Research Building, Room 814, Boston, MA 02115, USA.

[2]The State Key Laboratory of Experimental Hematology, Institute of Hematology and Blood Diseases Hospital, Chinese Academy of Medical Sciences and Peking Union Medical College, 288 Nanjing Road, Tianjin, 300020, China

[3]Cergentis BV, Yalelaan 62, 3584 CM Utrecht, the Netherlands.

*Correspondence should be addressed to: Hongbo.Luo@childrens.harvard.edu

†These authors have contributed equally to this work

A

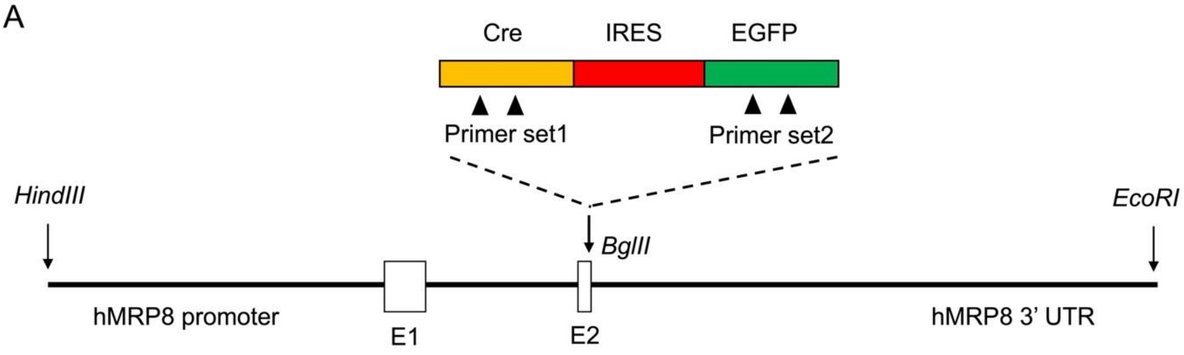

B

Primer set	Name	Direction	Binding position	Sequences
1	Cre	Reverse	330	AACCAGTGAAACAGCATTG
		Forward	408	GTTCGAACGCACTGATTTC
2	EGFP	Reverse	292	CGTCCTTGAAGAAGATGGT
		Forward	438	CAACAGCCACAACGTCTATA

Supplemental Figure 1. The structure of the hMRP8-Cre/ires-EGFP transgene and the primers used for TLA sequencing. (A) The 4.5 kb fragment of hMRP8 between *HindIII* and *EcoRI* cutting sites were used to generate the hMRP8-Cre/ires-EGFP transgene. A Cre/ires-EGFP cassette was inserted into the hMRP8 fragment at the *BglII* site between exons 2 and 3. (B) Sequences and binding positions of the two primer sets used for TLA sequencing targeting the Cre or EGFP region, respectively.

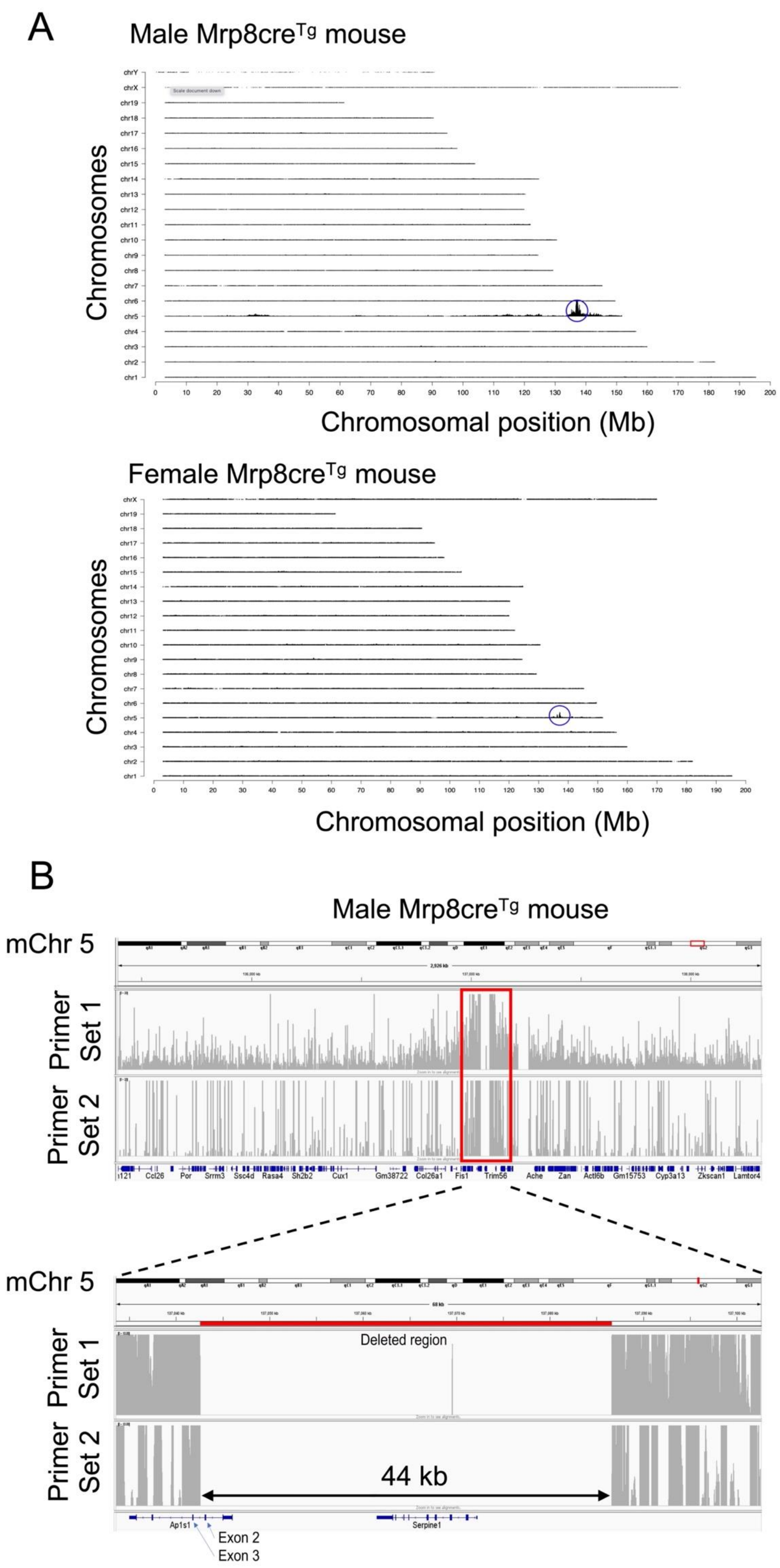

A
Male Mrp8cre^{Tg} mouse
Chromosomes
Chromosomal position (Mb)
Female Mrp8cre^{Tg} mouse
Chromosomes
Chromosomal position (Mb)
B
Male Mrp8cre^{Tg} mouse
mChr 5
Primer Set 1
Primer Set 2
Ccl26
Por
Srrm3
Ssc4d
Rasa4
Sh2b2
Cux1
Gm38722
Col26a1
Fis1
Trim56
Ache
Zan
Actl6b
Gm15753
Cyp3a13
Zkscan1
Lamtor4
mChr 5
Deleted region
Primer Set 1
Primer Set 2
44 kb
Ap1s1
Serpine1
Exon 2
Exon 3

Supplemental Figure 2. TLA sequencing shows the hMRP8-Cre/ires-EGFP transgene insertion site at chromosome 5 of the mouse genome. (A) Whole genome coverage plot of TLA sequencing of the male (upper panel) or female (lower panel) mice using primer set 1. Similar results were obtained with primer set 2. The blue circles highlight the TLA targeting area of primer set 1 on mouse Chr 5. (B) Magnified view of the blue circle area shows mouse Chr 5:135,000 -138,000 kb (upper panel) generated with primer sets 1/2. The red rectangle indicates a further magnified area of 137,030 – 137,100 kb (lower panel) exhibiting the 44 kb deletion on Chr 5 identified using both primer sets. Similar results were obtained in the female Mrp8creTg mouse.

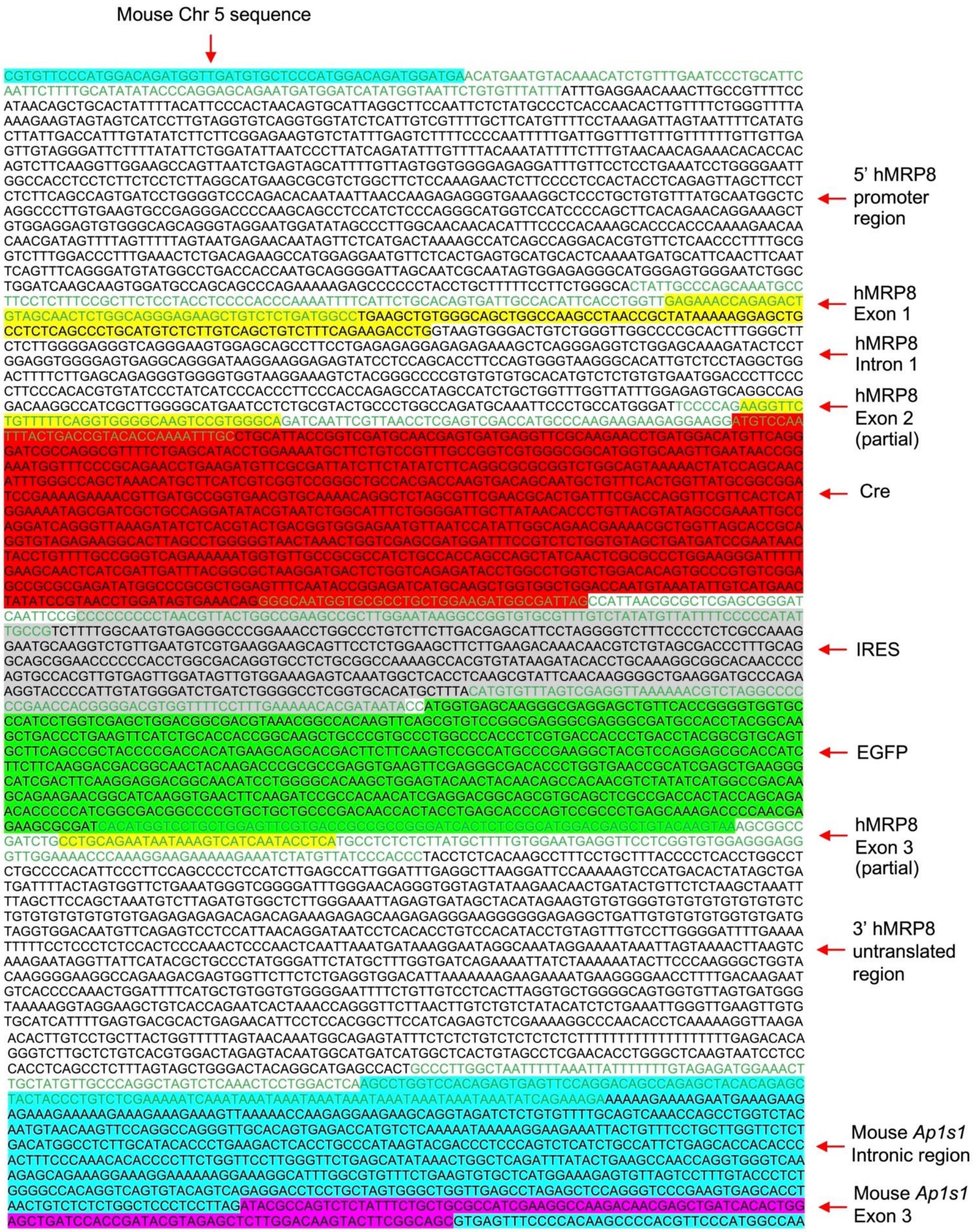
Mouse Chr 5 sequence
5' hMRP8 promoter region
hMRP8 Exon 1
hMRP8 Intron 1
hMRP8 Exon 2 (partial)
Cre
IRES
EGFP
hMRP8 Exon 3 (partial)
3' hMRP8 untranslated region
Mouse Ap1s1 Intronic region
Mouse Ap1s1 Exon 3

Supplemental Figure 3. Reconstructed hMRP8-Cre/ires-EGFP transgene sequence and its connecting sequences in the C57BL/6 mouse genome (chromosome 5). The hMRP8-Cre/ires-EGFP transgene sequence along with its flanking sequences on mouse Chr5 reconstructed based on the TLA sequencing results. Letters in green indicate the sequences confirmed by TLA sequencing. Letters in black are sequences BLASTed and mapped from reference sequences, including hMRP8, Cre, ires, EGFP, and part of the mouse *Ap1s1* gene sequence. Blue block: the mouse Chr 5 sequences; Yellow block: exons 1, 2, and 3 of hMRP8. Red block: the Cre recombinase sequence; Gray block: the IRES element sequence; Green block: the EGFP sequence; Pink block: exon 3 of mouse *Ap1s1*. Letters in green without other color codes are sequences from the cloning vectors. Sequence of the transgene has been uploaded to the online database GenBank for public access.

A

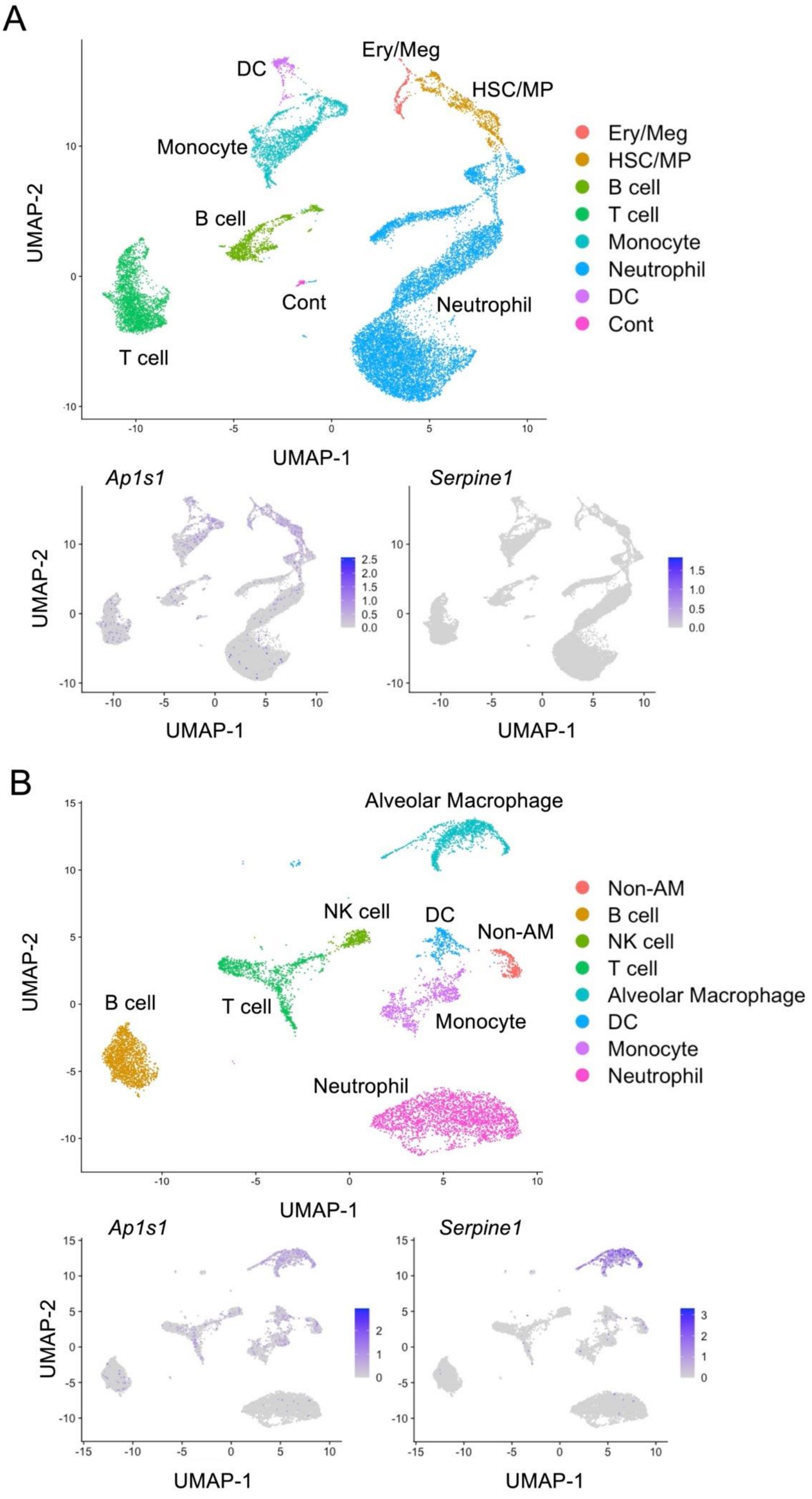

A
Ery/Meg
DC
HSC/MP
Monocyte
B cell
Cont
T cell
Neutrophil
UMAP-2
UMAP-1
Ery/Meg
HSC/MP
B cell
T cell
Monocyte
Neutrophil
DC
Cont
Ap1s1
Serpine1
B
Alveolar Macrophage
NK cell
DC
Non-AM
T cell
Monocyte
Neutrophil
Non-AM
B cell
NK cell
T cell
Alveolar Macrophage
DC
Monocyte
Neutrophil

Supplemental Figure 4. Single-cell sequencing data show the expression level of *Ap1s1* or *Serpine1* in different immune cells. (A) Single-cell sequencing data show low mRNA expression levels of both *Ap1s1* and *Serpine1* in mature neutrophils from the bone marrow, peripheral blood, and spleen (*28*). (B) High mRNA expression levels of *Ap1s1* and *Serpine1* are observed in alveolar macrophages but not pulmonary neutrophils. For both (A) and (B), the upper panels show the immune cell types while the lower panels show individual gene expression. *S100a8* is used as the neutrophil marker, while *Mrc1* is used as the macrophage marker. HSC: hematopoietic stem cell; MP: myeloid progenitor; Ery: erythrocyte; Meg: megakaryocyte; DC: dendritic cell; AM: alveolar macrophage; NK: natural killer; Cont: contaminating population.

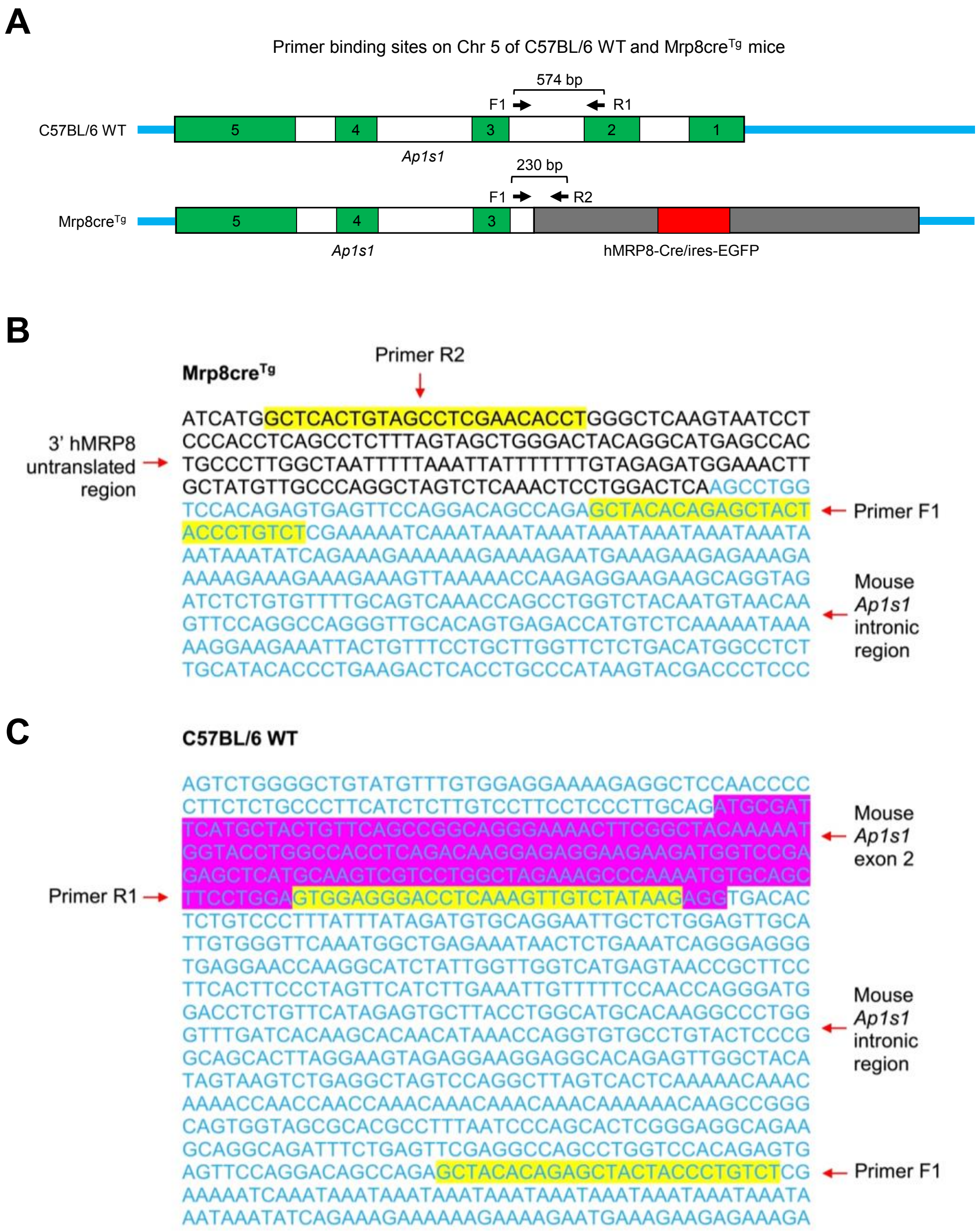

A
Primer binding sites on Chr 5 of C57BL/6 WT and Mrp8creTg mice
574 bp
F1
R1
C57BL/6 WT
5
4
3
2
1
Ap1s1
230 bp
F1
R2
Mrp8creTg
5
4
3
Ap1s1
hMRP8-Cre/ires-EGFP
B
Primer R2
Mrp8creTg
3' hMRP8 untranslated region
ATCATGGCTCACTGTAGCCTCGAACACCTGGGCTCAAGTAATCCT
CCCACCTCAGCCTCTTTAGTAGCTGGGACTACAGGCATGAGCCAC
TGCCCTTGGCTAATTTTTAAATTATTTTTTTGTAGAGATGGAAACTT
GCTATGTTGCCCAGGCTAGTCTCAAACTCCTGGACTCAAGCCTGG
TCCACAGAGTGAGTTCCAGGACAGCCAGAGCTACACAGAGCTACT
ACCCTGTCTCGAAAAATCAAATAAATAAATAAATAAATAAATAAATA
AATAAATATCAGAAAGAAAAAAGAAAAGAATGAAAGAAGAGAAAGA
AAAAGAAAGAAAGAAAGTTAAAAACCAAGAGGAAGAAGCAGGTAG
ATCTCTGTGTTTTGCAGTCAAACCAGCCTGGTCTACAATGTAACAA
GTTCCAGGCCAGGGTTGCACAGTGAGACCATGTCTCAAAAATAAA
AAGGAAGAAATTACTGTTTCCTGCTTGGTTCTCTGACATGGCCTCT
TGCATACACCCTGAAGACTCACCTGCCCATAAGTACGACCCTCCC
Primer F1
Mouse Ap1s1 intronic region
C
C57BL/6 WT
AGTCTGGGGCTGTATGTTTGTGGAGGAAAAGAGGCTCCAACCCC
CTTCTCTGCCCTTCATCTCTTGTCCTTCCTCCCTTGCAGATGCGAT
TCATGCTACTGTTCAGCCGGCAGGGAAAACTTCGGCTACAAAAAT
GGTACCTGGCCACCTCAGACAAGGAGAGGAAGAAGATGGTCCGA
GAGCTCATGCAAGTCGTCCTGGCTAGAAAGCCCAAAATGTGCAGC
TTCCTGGAGTGGAGGGACCTCAAAGTTGTCTATAAGAGGTGACAC
TCTGTCCCTTTATTTATAGATGTGCAGGAATTGCTCTGGAGTTGCA
TTGTGGGTTCAAATGGCTGAGAAATAACTCTGAAATCAGGGAGGG
TGAGGAACCAAGGCATCTATTGGTTGGTCATGAGTAACCGCTTCC
TTCACTTCCCTAGTTCATCTTGAAATTGTTTTTCCAACCAGGGATG
GACCTCTGTTCATAGAGTGCTTACCTGGCATGCACAAGGCCCTGG
GTTTGATCACAAGCACAACATAAACCAGGTGTGCCTGTACTCCCG
GCAGCACTTAGGAAGTAGAGGAAGGAGGCACAGAGTTGGCTACA
TAGTAAGTCTGAGGCTAGTCCAGGCTTAGTCACTCAAAAACAAAC
AAAACCAACCAACCAAACAAACAAACAAACAAAAAACAAGCCGGG
CAGTGGTAGCGCACGCCTTTAATCCCAGCACTCGGGAGGCAGAA
GCAGGCAGATTTCTGAGTTCGAGGCCAGCCTGGTCCACAGAGTG
AGTTCCAGGACAGCCAGAGCTACACAGAGCTACTACCCTGTCTCG
AAAAATCAAATAAATAAATAAATAAATAAATAAATAAATAAATAAATA
AATAAATATCAGAAAGAAAAAAGAAAAGAATGAAAGAAGAGAAAGA
Primer R1
Mouse Ap1s1 exon 2
Mouse Ap1s1 intronic region
Primer F1

Supplemental Figure 5. Targeting sites of the newly design primer set in Mrp8cre^{Tg} or C57BL/6 wildtype mice genome.

(A) Schematic to show the targeting sites of the newly designed primers, Forward 1 (F1), Reverse 1 (R1), and Reverse 2 (R2), highlighted in yellow blocks. **(B-C)** Primer sequences shown here are the reverse complements to the sequences shown in Figure 2. Primer F1 binds to a common sequence in mouse genome, the intronic region between *Ap1s1* gene exon 2 and exon 3; Primer R1 binds to *Ap1s1* gene exon 2, which generates a 574 bp PCR fragment with the wildtype allele (B). Primer R2 binds the hMRP8 3' untranslated region in Mrp8cre^{Tg} mouse genome, which generates a 230 bp fragment in PCR (A). Therefore, one band of 574 bp fragment indicates the wildtype mice while two bands of both fragments indicate the heterozygous Mrp8cre^{Tg} mice (Figure 2). Letters in black denote the hMRP8-Cre IRES/EGFP transgene sequence, and letters in blue denote the mouse genome sequences. Exon 2 of *Ap1s1* is highlighted in pink.
